# Optimizing sonication-assisted hydrodistillation of *Cinnamomum tamala* essential oil using response surface methodology and artificial neural network modeling

**DOI:** 10.1038/s41598-026-42869-2

**Published:** 2026-03-18

**Authors:** Parvej Hasan Jon, Jahid Hasan Shourove, Md. Kashem Ali, Oliur Rahman, Mostak Uddin Thakur, G. M. Rabiul Islam

**Affiliations:** 1 Department of Food Engineering and Tea Technology, Shahjalal University of Science and Technology, Sylhet, 3114 Bangladesh; 2 Department of Chemistry, University of Chittagong, Chittagong, 4331 Bangladesh

**Keywords:** Cinnamomum tamala, Sonication-assisted hydrodistillation, Essential oil, Response surface methodology, Artificial neural network, GC–MS analysis, Chemistry, Plant sciences

## Abstract

This study optimized the sonication-assisted hydrodistillation (SAHD) process for extracting essential oil (EO) from *Cinnamomum tamala* leaves, aiming to maximize yield and antioxidant activity. Both response surface methodology (RSM) and artificial neural network (ANN) models were used to predict extraction performance. The interpretability of the ANN model was enhanced using a neural interpretation diagram (NID), Olden’s algorithm, and sensitivity analysis, and it demonstrated higher accuracy and generalization than RSM. Under optimized conditions, the EO yield reached 1.67 ± 0.13%, with strong antioxidant activity indicated by a total phenolic content (TPC) of 79.24 ± 0.82 mg GAE/g and 81.54 ± 0.88% inhibition of the 2,2-diphenyl-1-picrylhydrazyl radical (DPPH). Residual analysis showed that both models satisfied key regression assumptions, including normality, independence, homoscedasticity, and lack of bias. Gas chromatography–mass spectrometry (GC–MS) analysis identified linalool (47.37%), eugenol (18.34%), and cinnamaldehyde (16.45%) as key constituents. Physicochemical characterization verified EO quality and stability. The integrated modeling approach provides a robust framework for enhancing EO extraction efficiency.

**Supplementary Information:**

The online version contains supplementary material available at 10.1038/s41598-026-42869-2.

## Introduction

In response to the global shift from synthetic chemicals toward sustainable organic substances, essential oils (EOs) have gained increasing interest as natural alternatives^1^. EOs are natural mixtures of volatile and non-volatile compounds found in various plant parts, mainly in the glandular trichomes of leaves and flowers^2^. EOs are abundant in bioactive compounds such as phenolics and terpenes and are valued for their strong antioxidant properties and wide array of applications in the cosmetic, pharmaceutical, and food industries^3–7^. As demand grows, efforts to enhance their yield and quality continue, with leaves generally yielding the highest oil content^2^.

*Cinnamomum tamala*, also known as Indian bay leaf or “tejpata”, is a notable medicinal plant belonging to the Lauraceae family, offering antimicrobial, anti-inflammatory, anti-diabetic, hypolipidemic, preservative and aromatic benefits^8–11^. Indigenous to southern Bangladesh and northeastern India^12^, its leaves are widely used as a culinary spice, although its EO holds considerable potential for industrial applications^7^. However, the EO industry faces challenges related to sustainability, quality control, and standardization, with low extraction yields being a major limitation that necessitates innovative extraction techniques^13^. Traditional methods such as hydrodistillation and steam distillation, though effective, often require significant energy input and extended processing times. Innovative methods such as solvent extraction, supercritical fluid extraction, and cold pressing have also been applied for EO extraction^14^. However, solvent extraction and supercritical fluid extraction face challenges due to their reliance on organic solvents and specialized equipment, respectively, while cold pressing has limited applicability beyond citrus fruits^15–17^. Hydrodistillation remains a well-established EO extraction method because of its simplicity, wide applicability and favorable consumer perception^18^. Among different techniques for bay leaf essential oil (BLEO) extraction, hydrodistillation has been reported to yield higher oil content compared to hydro-steam distillation, ohmic heating and microwave-assisted extraction^19^. Furthermore, the synergistic effects of ultrasound and hydrodistillation, known as sonication-assisted hydrodistillation (SAHD), have been established for enhanced EO extraction from a variety of plant matrices, including lavender, citrus, and ocimum plants, as well as plant parts like barks and buds^20–25^. However, Mohanty et al.^26^ recently reported the optimization of *C. tamala* essential oil extraction using microwave-assisted hydrodistillation, where rapid volumetric heating was identified as the primary mechanism for yield enhancement. In contrast, the present study employs sonication-assisted hydrodistillation, in which acoustic cavitation–induced micro-jetting and cell wall disruption govern mass transfer, enabling not only higher oil recovery but also improved preservation of phenolic compounds and antioxidant activity, underscoring the technological novelty and added value of the present study. Moreover, the application of SAHD to bay leaves remains relatively unexplored. SAHD typically reduces energy consumption, thereby contributing to Sustainable Development Goals (SDG) 12 by prioritizing energy efficiency and promoting sustainable practices^27–29^.

Although achieving a high yield is important, it should not overshadow the significance of producing high-quality EO while conserving energy resources^30^. Therefore, alongside yield optimization, ensuring superior EO quality is crucial to support the seamless transition from laboratory-scale research to large-scale industrial production. In this context, both response surface methodology (RSM) and artificial neural network (ANN) modeling can be used to comprehensively understand the SAHD process for EO extraction from bay leaves. RSM is a widely employed statistical approach for process optimization and is valued for its experimental efficiency, ease of implementation, and ability to provide interpretable mathematical relationships between process variables and responses^31^. It primarily involves designing experiments based on statistical principles, fitting empirical models to the obtained data, and using these models to predict optimal conditions^32^. An important advantage of RSM is its ability to quantify the individual and interactive effects of independent variables using analysis of variance (ANOVA), making it particularly useful for process interpretation and decision-making^31^. RSM has been extensively applied across diverse fields, including the food industry, to optimize processes, enhance product quality, and reduce costs^33,34^. Nevertheless, RSM may exhibit limitations when dealing with highly complex processes, as it relies on polynomial models that may not completely capture intricate non-linear interactions. In contrast, ANNs are data-driven computational models inspired by the structure and function of the human brain and are capable of learning complex non-linear relationships. ANNs predict responses based on training processes that adjust network weights to minimize prediction error^35^. Recent advancements in ANN applications for modeling complex biological and extraction systems have significantly increased their use in scientific research^36–39^. However, ANNs may sometimes underperform compared to RSM when applied to small datasets or when the relationships between process variables and responses are predominantly linear^40^. Therefore, employing both RSM and ANN in a comparative framework allows for the exploitation of the interpretability and simplicity of RSM alongside the strong predictive capability of ANN, providing a more robust and reliable understanding of the SAHD process.

To the best of our knowledge, no previous study has explored the optimization of the relatively novel SAHD technique for extracting EO from *C. tamala*, a widely cultivated species in Bangladesh. The novelty of this study lies in its multifaceted approach, employing RSM coupled with desirability function (RSM-DF), and ANN integrated with genetic algorithm (ANN-GA) to model and optimize the SAHD process with a focus on EO yield. This study also examined and optimized the effects of SAHD on the antioxidant properties of extracted EO. The robustness and reliability of the predictive models were confirmed through comprehensive residual and diagnostic analyses. Additionally, the EO obtained under optimal conditions was characterized using gas chromatography–mass spectrometry (GC–MS) and standard physicochemical assays. By identifying and modeling the optimal conditions for SAHD, this study can contribute to the advancement of sustainable extraction technologies and potentially support the industrial-scale production of high-quality BLEO, aligning with cleaner production goals.

## Materials and methods

### Chemical reagents

All chemicals and reagents used in this study were of analytical grade and employed without further purification. Folin-Ciocalteu reagent, 2,2-diphenyl-1-picrylhydrazyl (DPPH), and anhydrous sodium sulfate were purchased from Sigma-Aldrich (USA). Sodium carbonate (Na_2_CO_3_), iodine, potassium hydroxide (KOH), hydrochloric acid (HCl), phenolphthalein indicator, ethanol (C_2_H_5_OH), sodium hydroxide (NaOH), sodium thiosulfate (Na_2_S_2_O_3_), and starch solution were obtained from Merck (Germany). Distilled water was used for the preparation of all solutions.

### Sample collection and preparation

Bay leaves were purchased from a super shop located at the Madina market, Sylhet, Bangladesh. The plant sample was taxonomically authenticated based on the morphological characteristics of the leaves. The collected leaves were cleansed to remove surface dust and dried under ambient conditions (20–25 °C) in a shaded area of the laboratory for 5 days. Subsequently, the samples were oven-dried at 40 °C for 24 h, weighed, and further dried until a constant weight was achieved. The dried leaves were then chopped into sufficiently small and uniform pieces. Finally, the chopped samples were stored in airtight zipper bags, placed in sealed containers, and kept at 4 °C in a refrigerator (RT2ASKTS, Samsung, South Korea) until further experimentation.

### Sonication-assisted hydrodistillation (SAHD)

EO was extracted from bay leaves using a method adapted from Memarzadeh et al.^41^, with some modifications. Briefly, processed dried leaves were placed in a 500 mL round-bottom flask, and water was used as the carrier solvent to maintain the desired liquid-to-solid ratio. The mixture was then subjected to ultrasound pretreatment in an ultrasonic bath (FS-150 N, Shanghai Shengxi Supersonic Instrument Co., Ltd) operating at 20 kHz for various combinations of sonication time and power. After sonication, the flask was mounted on a Clevenger-type apparatus and heated using a 400 W electric heating mantle for hydrodistillation. The distillation time was recorded from the first appearance of droplets in the condensation column. The extracted EO was dried using anhydrous sodium sulfate to remove any residual moisture and stored at 4 °C for further analysis. The EO yield was calculated using the following Eq. (1):

1 $$Yield{\text{ }}(\% )={\text{ }}\frac{{{M_1}}}{{{M_2}}} \times 100$$

where M_1_ is the weight (g) of extracted EO, M_2_ is the weight (g) of the bay leaf sample.

### Assessment of total phenolic content (TPC)

The TPC of EO was determined using the Folin-Ciocalteu method as described by Kang et al.^42^, with minor modifications. Briefly, a 0.2 mL aliquot of EO dissolved in methanol (5 mg/mL) was mixed with 2.5 mL of Folin-Ciocalteu reagent (diluted 10-fold with deionized water). After allowing the mixture to react for 3 min at room temperature, 2.0 mL of 7.5% (w/v) sodium carbonate solution was added. The reaction mixture was then vortexed and incubated in the dark at room temperature for 60 min. Absorbance was measured at 765 nm against a distilled water blank using a UV-Vis spectrophotometer (UV2000, Shimadzu, Japan). A calibration curve was constructed using gallic acid standards at concentrations ranging 5–100 mg/mL, yielding the regression equation y = 0.0188x + 0.032 (R^2^ = 0.9984), where y represents absorbance and x denotes gallic acid concentration. The TPC was expressed as milligrams of gallic acid equivalents per gram of EO (mg GAE/g) and calculated using Eq. (2):

2 $$TPC{\text{ }}({\raise0.5ex\hbox{$\scriptstyle {mg{\text{ }}GAE}$}\kern-0.1em/\kern-0.15em\lower0.25ex\hbox{$\scriptstyle {g{\text{ }}EO}$}})=\frac{{Abs - b}}{m} \times \frac{d}{w}$$

where, *Abs* is the absorbance at 765 nm, *b* is the y-intercept, *m* is the slope, *d* is the dilution factor, and *w* is the EO weight in gram.

### Assessment of 2,2-diphenyl-1-picrylhydrazyl (DPPH) scavenging activity

The antioxidant capacity of BLEO was measured in terms of % DPPH inhibition according to the methods of Brand-Williams et al.^43^, with slight modifications. Briefly, 50 µL of a methanolic solution of BLEO (100 µg/mL) was mixed with 2.0 mL of a 6 × 10^− 5^ M methanolic DPPH solution. The mixture was vortexed at 2500 rpm for 1 min to ensure thorough mixing and then incubated in the dark at room temperature for 30 min to allow the reaction to proceed. Absorbance was measured at 517 nm using a UV-Vis spectrophotometer. A blank control containing methanol instead of BLEO was used as the reference. The percentage inhibition of DPPH radical scavenging activity was calculated using Eq. (3):

3 $$Inhibition{\text{ }}(\% ){\text{ }}={\text{ }}\frac{{{A_b} - {A_s}}}{{{A_b}}} \times 100$$

where A_b_ and A_s_ are respectively the absorbance of the control and the test solutions at 517 nm.

### Response surface methodology (RSM) modeling

A rotatable central composite design (CCD) (k = 6) with a desirability function was employed to optimize the EO yield using the SAHD technique. The study focused on four key process variables: liquid-to-solid ratio (LSR, 8:1–12:1), sonication power (SP, 80–120 W), sonication time (ST, 20–40 min), and hydrodistillation time (HT, 120–180 min). The evaluated responses were EO yield, TPC, and DPPH radical scavenging activity. The ranges of the factors were determined based on preliminary single-factor experiments. In the CCD, process variables were examined at five coded levels (− α, − 1, 0, + 1, +α), where 0 represents the central point, + 1 and − 1 correspond to the highest and lowest levels within the investigated range, respectively. The axial distance (α), which depends on the number of factors (*n* = 4), was set at 2 to ensure rotatability. Rotatability ensures a uniform variance of the predicted response prediction at all points equidistant from the design center, thereby improving the reliability of the optimization process. The total number of experimental runs for CCD was calculated using the following Eq. (4):

4 $$N={2^k}+2k+{n_0}$$

where k is the number of factors, *2*^*k*^ is the number of factorial points, *2k* represents the axial points, and *n*_*0*_ is the number of center points.

The design matrix of this study from the rotatable CCD generated 30 experimental runs with 6 center points. Table 1 presents these experimental runs along with their corresponding responses.

**Table 1:** Rotatable central composite design (CCD) for the EO extraction using SAHD.

Run	Liquid-solid ratio (mL/g)	Sonication power (Watt)	Sonication time (min)	Hydro-distillation time (min)	Yield (%)	TPC (mg GAE/g)	DPPH inhibition (%)
1	10	120	30	180	0.98	63.91	70.19
2	10	200	30	120	1.13	66.71	66.17
3	12	160	20	150	0.71	76.69	78.55
4	10	120	30	60	0.54	47.81	42.59
5	8	160	20	150	0.98	50.21	53.14
6	12	80	20	150	0.88	72.05	70.19
7	10	120	30	120	1.76	77.82	81.43
8	12	80	40	90	0.83	54.37	61.24
9	10	120	10	120	0.85	64.22	68.73
10	12	160	40	90	1.11	59.81	66.71
11	10	120	30	120	1.67	75.38	75.17
12	8	80	20	90	0.56	51.48	38.29
13	14	120	30	120	0.93	74.53	74.33
14	8	80	20	150	0.97	54.63	49.83
15	10	120	30	120	1.66	73.28	71.28
16	10	120	50	120	1.18	66.89	69.91
17	10	120	30	120	1.68	75.28	75.13
18	6	120	30	120	0.99	41.97	38.82
19	12	80	20	90	0.86	65.17	58.87
20	8	80	40	150	1.32	52.61	54.28
21	10	120	30	120	1.64	79.84	77.86
22	10	40	30	120	1.11	52.10	55.19
23	8	80	40	90	0.69	49.98	47.57
24	8	160	40	150	1.34	59.84	59.84
25	12	160	40	150	1.09	69.84	72.31
26	12	160	20	90	0.94	69.83	66.16
27	8	160	40	90	0.96	58.31	58.73
28	10	120	30	120	1.67	74.28	80.12
29	12	80	40	150	1.14	70.01	69.43
30	8	160	20	90	0.78	55.43	59.47

After experiments, a quadratic model was developed to fit the response data. The optimum value of the responses was measured using the following second order polynomial equation as follows (Eq. 5):

5 $$Y={\beta _0}+\sum\limits_{{i=1}}^{k} {{\beta _i}{I_i}} +\sum\limits_{{i=1}}^{{k - 1}} {\sum\limits_{{j=i+1}}^{k} {{\beta _{ij}}{I_i}{I_j}} } +\sum\limits_{{i=1}}^{k} {{\beta _{ii}}{I_i}^{2}}$$

where *Y* is the predicted response, *β*_*0*_ is the constant coefficient, *β*_i_, *β*_*ij*_, *and β*_*ii*_ are respectively the linear, interaction and quadratic coefficients, *I*_*i*_ and *I*_*j*_ are the independent variables, and k is the number of the independent variables. Larger coefficients indicate a stronger influence, while negative coefficients signify an inverse relationship. To visualize the response surface, 3D plots were generated.

### Artificial neural network (ANN) modeling

In this study, an ANN model was developed utilizing the experimental data generated from the CCD. Neural networks are well-suited to model intricate and dynamic biological systems^44–46^. Given the complex nature of SAHD, a multi-layered perceptron (MLP) architecture was selected for its capability to interpret non-linear processes^47,48^. Hyperparameter tuning was performed to optimize key ANN parameters, including weight decay, the number of hidden neurons, and the activation function. The optimal configuration, selected based on by the coefficient of determination (R^2^) and root mean square error (RMSE), consisted of a weight decay value of 0.001, eight hidden neurons, and a logistic activation function. Backpropagation was employed as the learning algorithm of the ANN model for its relative ease, flexibility, and proven effectiveness^49–51^. To validate the model and reduce the risk of overfitting, k-fold cross validation (k = 10) was carried out. In addition, the ANN model was evaluated using an independent test dataset (*n* = 9) comprising of axial, central, and factorial points from the CCD design matrix. For the ANN model interpretation, the neural interpretation diagram (NID)^32,52,53^, Olden’s algorithm for assessing relative variable importance^54^ and Lek’s profile method^55^ were applied. Additionally, residual analysis was conducted to evaluate the underlying model assumptions includinghomoscedasticity, independence, normality, and zero mean of errors. Residual distribution was evaluated using Shapiro-Wilk’s test, normal Q-Q plots, and histograms. The independence of residuals was assessed through Durbin-Watson’s test, autocorrelation plots, and Pearson and Spearman correlation coefficients. Residual homoscedasticity was determined using Levene’s test and scale-location plots.

### Characterization of the extracted EO using GC–MS

The chemical composition of the extracted EO was analyzed using GC–MS (Shimadzu QP-2000, Japan). For this analysis, an Ulbon HR-1 fused silica capillary column with dimensions of 50 m × 0.25 mm and a film thickness of 0.25 μm was selected. The oven temperature was initially set at 50 °C for 4 min, followed by a gradual increase to 260 °C at a rate of 3 °C per min, where it was then maintained for an additional 5 min. Helium served as the carrier gas at a flow rate of 1.3 mL/min in split mode. The injector and detector temperatures were maintained at 230 °C and 270 °C, respectively. All prepared samples (10% v/v in hexane) were injected in a uniform volume of 0.1 µL with a split ratio of 1:30. Mass spectrometric detection was carried out using electron ionization (EI) at 70 eV. The Kovats retention index (KI), Wiley Registry, and previous literature^56–58^ were used to identify each EO compound. The KI values were calculated and compared against a standard series of alkanes (C8–C40) for confirmation. The KI was calculated using the following Eq. (6):

6 $$KI=100 \times \left[ {n+\frac{{tr(unknown) - t{r_n}}}{{t{r_N} - t{r_n}}}} \right]$$

where KI is the Kovats retention index, n is the smaller carbon number in the n-alkane, N is the number of carbon atoms in the larger n-alkane, and tr is the retention time.

### Analyses of physico-chemical properties

The physico-chemical properties of the EO obtained under optimal conditions were evaluated using established AOAC analytical techniques^59^. Color and odor were analyzed through visual and sensory assessments to provide preliminary insights. Specific gravity was measured using a mass over volume pycnometer^60^. The chemical properties, including iodine value, saponification value, acid value, and ester value, were determined according to the protocols described by Ibrahim et al.^61^.

### Statistical analyses

The CCD experimental design was carried out using Design Expert v. 12 software. RSM models were fitted and visualized in R/RStudio, utilizing packages such as “rsm,” “ggplot2,” “plotly,” and “gridextra”, etc. The ANN model training and testing were also performed in R statistical software, along with the plotting of the NID, calculation and visualization of Olden’s algorithm, and generation of Lek’s profile using packages like “nnet,” “caret,” “lattice,” and “NeuralNetTools.” Calculations were performed in Microsoft Excel and RStudio. All experiments were conducted in triplicate to ensure reliability and reproducibility.

## Results and discussion

### BLEO extraction overview

Table 1 presents the experimental design matrix, illustrating the influence of varying SAHD parameters on the extraction yield, TPC, and DPPH radical scavenging activity of BLEO. The extraction yield varied markedly across the experimental conditions, ranging from 0.54% to 1.76%, representing more than a threefold increase depending on the combination of process factors. These findings highlight the significant influence of ultrasound and associated process variables on oil recovery efficiency.

The maximum yield obtained in this study (1.76%) notably exceeds those reported using conventional hydrodistillation methods, which typically range between 0.25% and 0.60%^9,62,63^. Similarly, Řebíčková et al.^64^ reported yields of 0.95% from hydrodistillation and 0.79% from steam distillation of Brazilian bay leaves. The comparative analysis of extraction methods highlights the efficiency of sonication-assisted hydrodistillation for BLEO recovery. In the present study, SAHD of *C. tamala* leaves yielded 1.67% essential oil within approximately 124 min, demonstrating a clear reduction in processing time relative to conventional hydrodistillation^26,64–66^. A recent study on the same plant material showed lower yields with longer durations, such as 1.40% in 240 min using hydrodistillation and 1.55% in 85 min using microwave-assisted hydrodistillation, indicating that ultrasound integration provides a favorable balance between extraction efficiency and time^26^.

When compared with other botanical sources, yield variability is strongly influenced by plant matrix, oil localization, and cell wall structure. For example, *Cinnamomum cassia* bark consistently exhibits higher oil recovery (up to ~ 2.7%) due to its resin-rich structure, with process intensification techniques (sonication or microwave assistance) markedly reducing extraction time from 180 min to as low as 30 min^65,66^. In contrast, low-oil matrices such as *Carex meyeriana* required prolonged hydrodistillation (9 h) with minimal yield (0.13%), underscoring the limitations of conventional methods for structurally rigid or low-volatile plant tissues^67^. SAHD also demonstrated competitive performance relative to other intensification technologies across diverse plant materials. For citrus peels and bitter orange, ultrasound reduced extraction time while moderately improving yield^68^, whereas for clove buds—naturally rich in essential oil—SAHD achieved very high recovery (15.23%) within only 50 min^21^. Ohmic heating and combined ultrasonic/microwave systems similarly shortened processing times, but often require more complex instrumentation and higher energy input^25,69^. Although ohmic heating yielded up to 1.35% in a study by Tunç and Koca^69^, the SAHD approach employed here achieved superior performance. This enhancement may be attributed to the mechanical effects of acoustic cavitation, which promote more efficient cell wall disruption and facilitate solvent penetration.

In terms of bioactivity, BLEO exhibited TPC values ranging from 41.97 to 79.84 mg GAE/g and DPPH radical scavenging activity ranging from 38.29% to 81.43%. These results are consistent with the findings of Rincón et al.^70^, who reported a TPC of 83.41 mg GAE/g and antioxidant activity of 74.73% in ethanolic BLEO extracts. A comparison with previously reported related extraction methods is summarized in Table 2.

**Table 2:** Comparative analysis of essential oil extraction techniques across diverse plant materials.

Method	Plant material	EO yield	Extraction time	References
Sonication-assisted hydrodistillation	*Cinnamomum tamala* leaves	1.67%	~ 124 min	Present study
Hydrodistillation, steam distillation	*Laurus nobilis* leaves	0.95%, 0.79%	270 min, 280 min	ADDIN EN.CITE ^64^
Hydrodistillation, sonication-assisted hydrodistillation, microwave-assisted hydrodistillation	*Cinnamomum cassia* barks	2.67%, 2.70%, 2.72%	180 min, ~ 62 min, ~ 30 min	^65^
Hydrodistillation, sonication-assisted hydrodistillation	*Cinnamomum cassia* barks	1.68%, 2.11%	120 min, 45 min	^66^
Hydrodistillation	*Carex meyeriana*	0.13%	9 h	^67^
Hydrodistillation, sonication-assisted hydrodistillation	Bitter orange peel	0.65%, 0.90%	4.92 h, 4.72 h	^68^
Sonication-assisted hydrodistillation	Clove buds	15.23%	50 min	^21^
Hydrodistillation, microwave-assisted hydrodistillation	*Cinnamomum tamala* leaves	1.4%, 1.55%	240 min, 85 min	^26^
Hydrodistillation	*Cymbopogon flexuosus*	1.04%	-	^78^
Ohmic heating assisted hydrodistillation	Cinnamon and bay leaf	3.98%, 1.36%	119.9 min, 120 min	^69^
Ultrasonic/microwave assisted hydrodistillation, hydrodistillation, microwave- assisted hydrodistillation, solvent extraction	*Citrus* *medica*	0.60%, 0.52%, 0.58%, 0.70%	15.5 min, 1.5 h, 15 min, 90 min	^25^

**Fig. 1:**
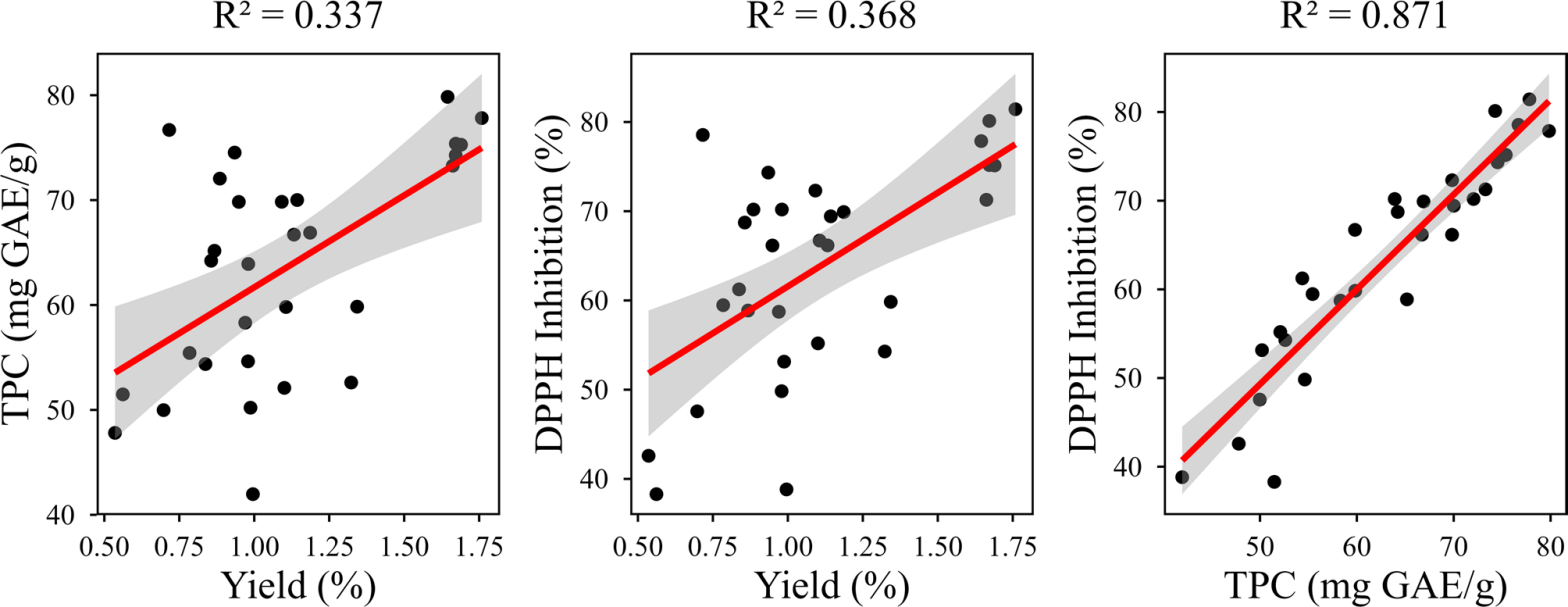
Correlation plot between responses.

A strong positive correlation (R^2^ = 0.871) was observed between TPC and DPPH scavenging capacity (Fig. 1), underscoring the pivotal role of phenolic compounds in contributing to antioxidant activity. Phenolics, including flavonoids and phenolic acids, are known to neutralize free radicals through the donation of hydrogen atoms from their hydroxyl groups, forming stable intermediates that inhibit oxidative processes^71–73^. This correlation reinforces the notion that a higher concentration of phenolic constituents enhances the radical scavenging efficiency of BLEO.

### RSM modeling and parameter interactions

RSM was employed to evaluate the effects of SAHD parameters on BLEO yield, TPC, and DPPH radical scavenging activity. The statistical adequacy of the developed models was validated through ANOVA, with all three response models found to be highly significant (*p* < 0.05), as shown in Table 3. The non-significant lack-of-fit values (*p* > 0.05) further confirmed that the models were suitably fitted to the experimental data^32^. The R^2^ values for the RSM models in predicting yield, TPC, and DPPH inhibition were 0.987, 0.971, and 0.960, respectively, demonstrating strong model performance. Moreover, the minimal difference observed between R^2^ and adjusted R^2^ values further supports the reliability and close correlation between the models and the experimental data.

**Table 3:** Analysis of variance (ANOVA) of the RSM model for the EO yield.

	Yield		TPC		DPPH inhibition	
	Sum of square	*P*-value	Sum of square	*P*-value	Sum of square	*P*-value
Model	3.49	< 0.0001	3090.02	< 0.0001	4010.62	< 0.0001
I_1_-Liquid-solid ratio	0.002	0.477	1209.84	< 0.0001	1557.35	< 0.0001
I_2_-Sonication power	0.048	0.004	144.45	0.001	316.61	0.0004
I_3_-Sonication time	0.236	< 0.0001	9.86	0.309	13.46	0.359
I_4_-Hydrodistillation time	0.343	< 0.0001	226.32	0.0001	465.78	< 0.0001
I_1_I_2_	0.009	0.155	0.016	0.965	18.51	0.284
I_1_I_3_	0.001	0.544	93.61	0.005	35.31	0.146
I_1_I_4_	0.130	< 0.0001	87.05	0.007	37.42	0.135
I_2_I_3_	0.006	0.230	9.00	0.331	14.19	0.346
I_2_I_4_	0.055	0.002	14.25	0.225	39.03	0.128
I_3_I_4_	0.056	0.002	20.61	0.149	3.34	0.644
I_1_^2^	0.896	< 0.0001	504.06	< 0.0001	724.56	< 0.0001
I_2_^2^	0.801	< 0.0001	438.45	< 0.0001	464.10	< 0.0001
I_3_^2^	0.821	< 0.0001	166.07	0.0006	104.66	0.018
I_4_^2^	1.17	< 0.0001	654.37	< 0.0001	737.66	< 0.0001
Residual	0.064		133.70		225.72	
Lack of fit	0.057	0.069	104.39	0.272	156.23	0.477
R-squared	0.987		0.971		0.960	
Pure error	0.007		29.32		69.49	
Cor total	3.55		3223.73		4236.34	

For BLEO yield, all factors were significant except for LSR, with interactions LSR–SP, LSR–ST, LSR–SP, and SP–ST having p-values of 0.477, 0.155, 0.544, and 0.230, respectively. For TPC and DPPH inhibition, all linear factors, except ST, were significant. However, for BLEO yield, although linear term of LSR was insignificant, its interactive effect with HT was found to significantly affect yield. HT also had interactive effects with SP and ST denoted by I_2_I_4_ and I_3_I_4_. TPC only had I_1_I_3_ and I_1_I_4_ as their statistically significant interactive terms. All quadratic terms in the three models were significant, suggesting a non-linear relationship in which changes in factors lead to a peak in responses, followed by diminishing returns. This also implies the presence of an optimum condition within our experimental domain, with no further adjustments required^27,31,74^. Based on these results, three second order polynomial quadratic regression models were developed as follows (Eqs. (7–9)):

7 $$\begin{aligned} Yield & = + 1.69 - 0.009I_{1} + 0.045I_{2} + 0.099I_{3} + 0.12I_{4} - 0.025I_{1} I_{2} - 0.010I_{1} I_{3} - 0.090I_{1} I_{4} \\ & \;\;\;\; + 0.020I_{2} I_{3} - 0.059I_{2} I_{4} + 0.059I_{3} I_{4} - 0.18I_{1}^{2} - 0.17I_{2}^{2} - 0.17I_{3}^{2} - 0.21I_{4}^{2} \\ \end{aligned}$$

8 $$\begin{aligned} TPC & = + 75.98 - 7.10I_{1} + 2.45I_{2} - 0.64I_{3} + 3.07I_{4} - 0.032I_{1} I_{2} - 2.42I_{1} I_{3} + 2.33I_{1} I_{4} \\ & \;\;\;\; + 0.75I_{2} I_{3} - 0.94I_{2} I_{4} + 1.14I_{3} I_{4} - 4.29I_{1}^{2} - 4.00I_{2}^{2} - 2.46I_{3}^{2} - 4.88I_{4}^{2} \\ \end{aligned}$$

9 $$\begin{aligned} Inhibition & = + 76.83 + 8.06I_{1} + 3.63I_{2} + 0.75I_{3} + 4.41I_{4} - 1.08I_{1} I_{2} - 1.49I_{1} I_{3} + 1.53I_{1} I_{4} \\ & \;\;\; - 0.94I_{2} I_{3} - 1.56I_{2} I_{4} - 0.46I_{3} I_{4} - 5.14I_{1}^{2} - 4.11I_{2}^{2} - 1.95I_{3}^{2} - 5.19I_{4}^{2} \\ \end{aligned}$$

### Effect of process parameters on responses

**Fig. 2:**
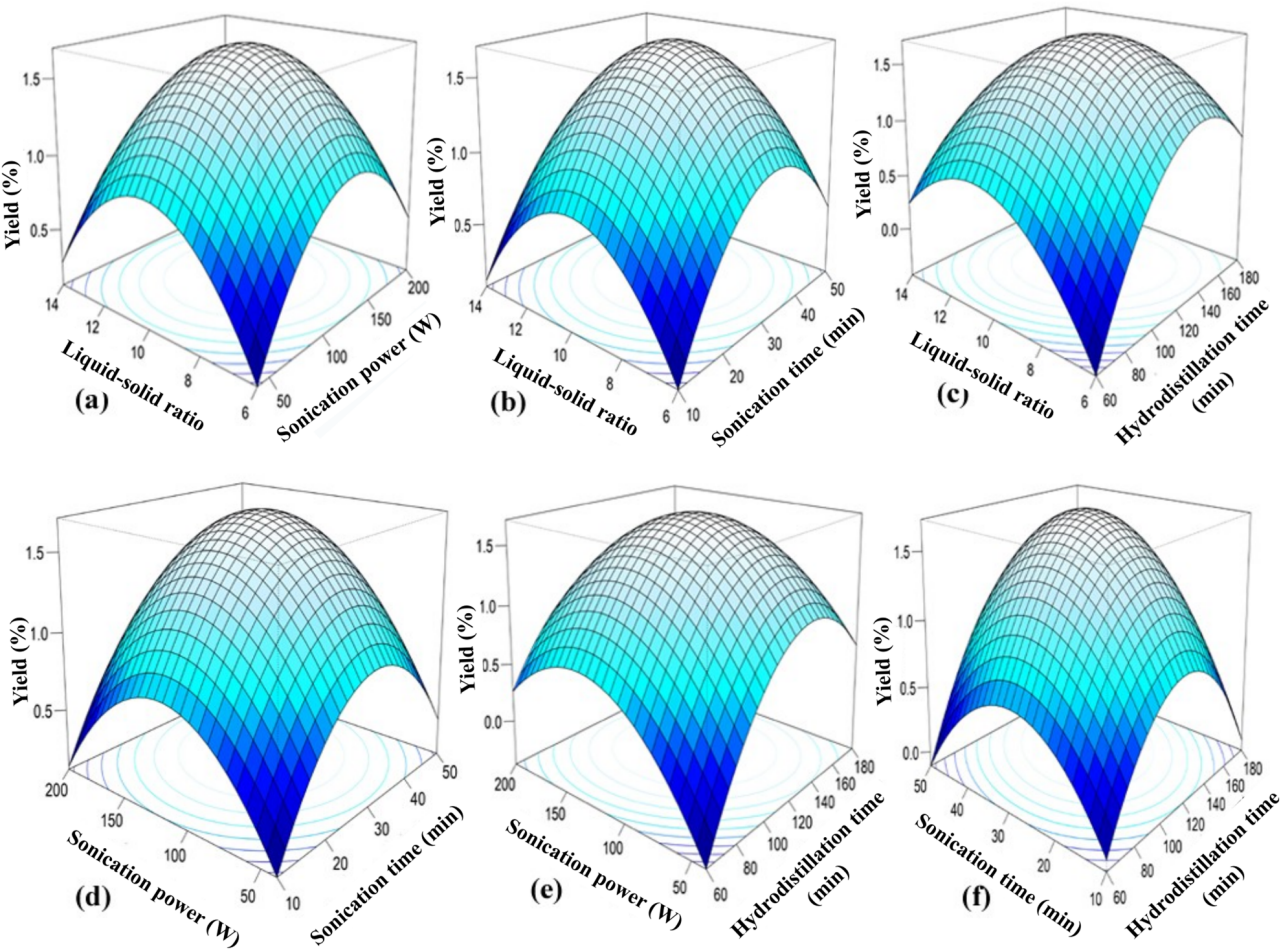
RSM 3D plots for EO yield.

**Fig. 3:**
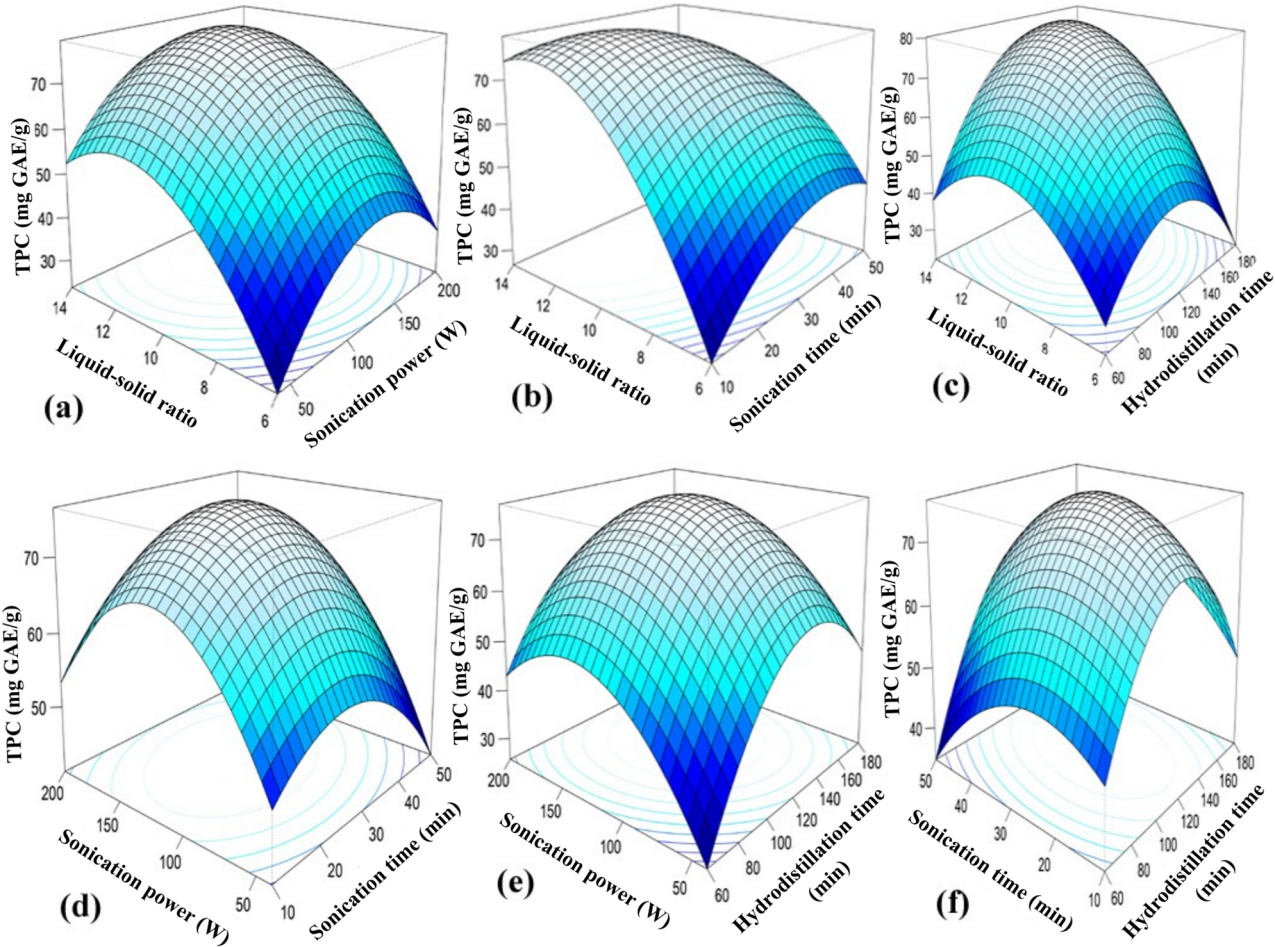
RSM 3D plots for EO TPC.

**Fig. 4:**
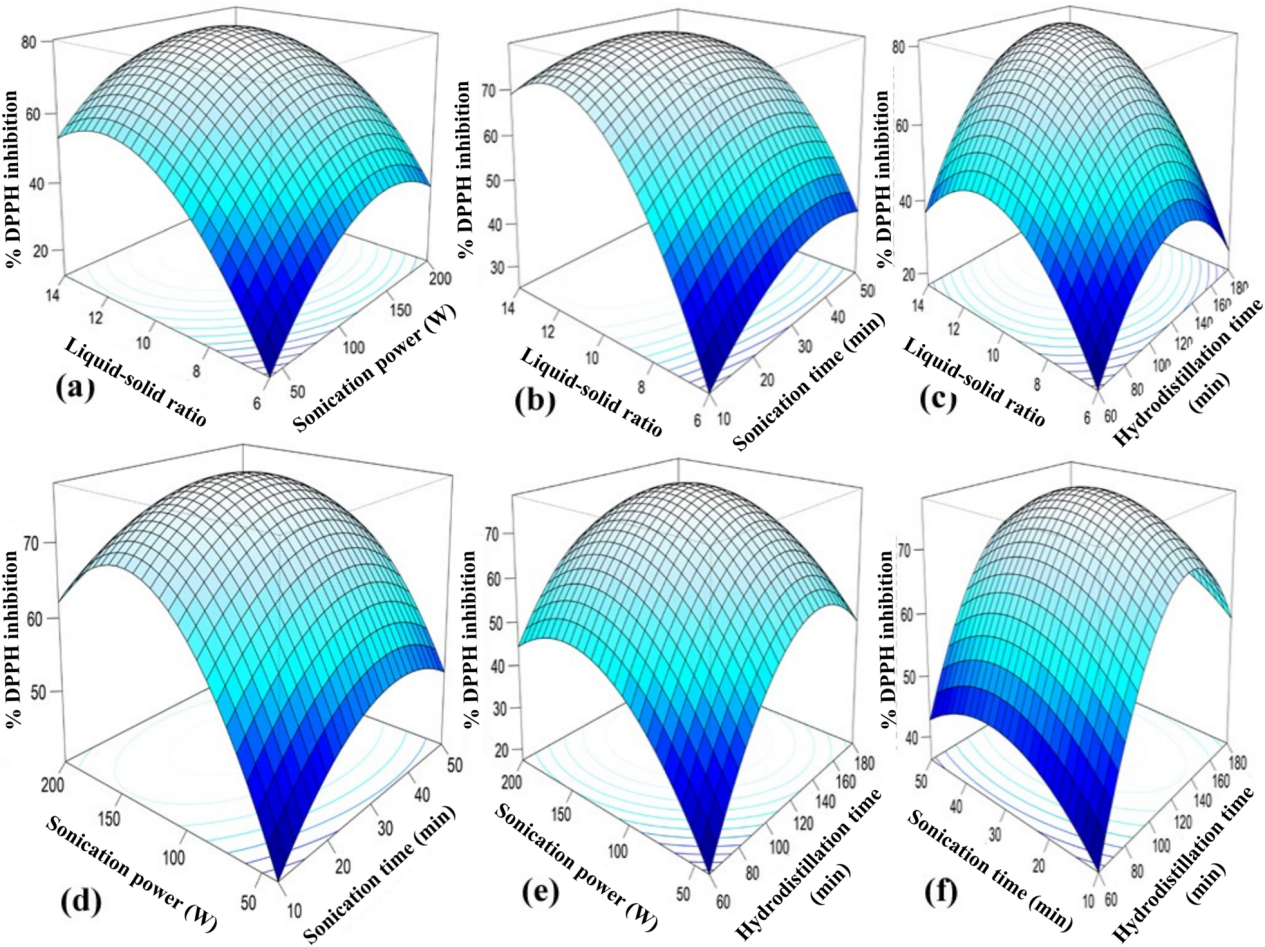
RSM 3D plots for EO DPPH inhibition.

The CCD captures both linear and quadratic effects of the variables, including axial (α) points extending beyond the factorial ranges. The 3D response surface plots for BLEO yield, TPC, and DPPH radical scavenging activity (Figs. 2, 3 and 4) of SAHD process exhibit similar trends, with clear maxima observed at intermediate levels of the process parameters. At lower levels of LSR, SP, ST, and HT, extraction efficiency is limited due to insufficient solvent penetration, reduced cavitation intensity, and inadequate disruption of plant cellular structures. In contrast, excessively high levels of these parameters may result in cavitation shielding, solvent saturation, prolonged thermal exposure, or degradation of heat-sensitive bioactive compounds, thereby reducing essential oil yield and quality. Consequently, the optimal extraction conditions occur within the mid-range of the tested variables, where enhanced mass transfer and cell wall disruption are achieved without compromising the chemical integrity of the extracted essential oil, phenolic content, and antioxidant activity. These results indicate the presence of optimal extraction conditions within the tested ranges, emphasizing the need for balanced process parameters to maximize extraction efficiency.

#### Liquid-solid ratio (LSR)

The LSR significantly influenced the extraction yield, TPC, and DPPH radical scavenging activity of BLEO, with all responses peaking at a moderate LSR of approximately 10:1 (Figs. 2a–c, 3a–c and 4a–c). As LSR increased from 6:1 to 10:1, the BLEO yield improved due to enhanced solute-solvent interactions and more effective mass transfer; however, further increases beyond 10:1 led to a marked decline in efficiency. In general, increasing the LSR within an optimal range enhances the solubility of target compounds, thereby improving extraction yield^75^. However, at lower ratios (6:1 or 8:1), limited solvent availability likely restricted diffusion and hindered extraction, potentially leading to early thermal degradation of sensitive compounds^76^. On the other hand, excessively high LSRs (12:1 and 14:1) may dilute solute concentrations, reducing the mass transfer driving force and spreading ultrasonic energy over a larger volume, thereby weakening cavitation effects and lowering recovery of EO and bioactives^66,77,78^. Although water is essential in hydrodistillation to prevent thermal degradation and support volatilization, using too much can increase energy consumption, extend extraction time, and complicate solvent recovery^79^. These observations align with previous findings in bitter leaf EO extraction^80^, and reinforce the importance of optimizing LSR to balance extraction kinetics, solvent efficiency, and compound stability. Nevertheless, Chen et al.^66^ reported a lower optimal LSR of 6:1 for *C. cassia* bark, highlighting that the optimal liquid-solid ratio is strongly influenced by both plant material characteristics and extraction conditions. Bark tissues generally possess a denser and more rigid structure with lower surface area and different oil localization compared to leaf matrices, which can alter solvent penetration and mass transfer behavior. In contrast, bay leaves (*C. tamala*) have a comparatively softer and more porous cellular structure, requiring a higher solvent volume to ensure effective swelling, cavitation-assisted cell disruption, and efficient solute diffusion under sonication-assisted hydrodistillation. Additionally, differences in extraction technique, energy input, and thermal exposure further contribute to variations in the optimal LSR reported across studies^26,65^.

#### Sonication power (SP)

The influence of SP on BLEO yield, TPC, and DPPH radical scavenging activity is shown in Figs. 2a, d and e and 3a, d and e; and 4a, d, e. At a fixed LSR (10:1), ST (40 min), and HT (120 min), all three responses increased with rising SP, peaking around 130 W. This enhancement is attributed to intensified cavitation at moderate SP levels, where the rapid collapse of microbubbles generates localized high-pressure and shear forces, disrupting plant cell walls and promoting the release of bioactive compounds^81,82^. At low SP levels (40–80 W), insufficient energy fails to produce adequate cavitation, limiting extraction efficiency. In contrast, excessive SP (> 130 W) may cause over-cavitation, generating free radicals and localized overheating, which can degrade thermolabile compounds and reduce EO yield and bioactivity^66,83^. These findings are in agreement with prior studies on SAHD of cinnamon bark^66,84^, highlighting that a moderate SP balances mechanical disruption and compound stability for optimal extraction performance.

#### Sonication time (ST)

ST proved to be another critical parameter that affected the extraction efficiency. RSM 3D plots showed that as the ST increased from 10 to 30 min, the BLEO yield (Fig. 2b, d and f), TPC (Fig. 3b, d and f), and DPPH radical scavenging activity (Fig. 4b, d and f) increased; however, they gradually declined when the ST was further increased from 30 to 50 min. Prolonged exposure to sonication action may raise the cell surface temperature, negatively affecting EO content. Therefore, after maximum extraction was achieved, longer sonication duration was redundant. Ultrasound energy expenditure can be minimized through precise control of the sonication process duration as these two factors inherently influence each other^85^. Therefore, a moderate ST is essential to balance the effective extraction without compromising the integrity of the BLEO. Our findings of the effect of ST are in line with Chen et al.^66^, Mollaei et al.^83^, Bahmani et al.^85^.

#### Hydrodistillation time (HT)

Figures 2c and e–f and 3c and e–f; and 4c, e–f showed a HT of approximately 120 min was optimal for maximizing EO yield, TPC, and DPPH radical scavenging activity from bay leaf. Increasing HT generally enhanced EO yields, aligning with findings by Mainya et al.^86^ and Yingngam and Brantner^87^, who observed similar trends in EO extraction through hydrodistillation. However, extending HT beyond 150 min resulted in a plateau effect, with no further increase in yields. Additionally, shorter HT ranging from 60 to 90 min, likely resulted in insufficient extraction of EO compounds, resulting in lower yields. This observation aligns with Cui et al.^67^, who reported that prolonged HT did not significantly improve EO yield. While traditional hydrodistillation methods often require extended periods (180–280 min) to maximize BLEO yield, as reported in previous studies^64,88^, the incorporation of sonication in the present investigation reduced extraction time substantially to 120 min while achieving comparable or higher yields.

### ANN model fitting

Due to the complex and non-linear nature of SAHD process variables, an ANN model was employed as an advanced mathematical tool to optimize BLEO yield, TPC, and DPPH assays. Its performance, evaluated through statistical parameters (Table 4), demonstrated its effectiveness in predicting extraction outcomes. Evaluation metrics presented a high degree of agreement between the model’s predictions and the observed data, as evidenced by R^2^ and adjusted R^2^ values ranging from 0.985 to 0.999 for all three responses. RMSE for BLEO yield, TPC and DPPH radical scavenging activity were 0.004, 0.127, and 1.31, respectively. Additionally, mean absolute error (MAE) was calculated to be similarly low, at 0.003 for yield, 0.096 for TPC and 1.11 for DPPH inhibition. A k-fold cross-validation was applied to the ANN model to mitigate “overfitting”, a common issue where the neural network model performs well on training data but fails to generalize to unseen data, resulting in high error rates^89^. The model’s generalizability was further evaluated using an independent dataset comprising central, axial, and factorial points (runs 2, 3, 6, 7, 9, 16, 20, 22, and 30) (Supplementary Table 1). The test dataset showed R^2^ and adjusted R^2^ values ranging 0.980–0.999 and 0.959–0.999, respectively (Supplementary Table 2), strongly confirming the ANN model’s reliability in predicting unseen data.

**Table 4:** Different performance parameters for RSM and ANN model.

Performance parameters	Yield		TPC		DPPH inhibition	
	RSM	ANN	RSM	ANN	RSM	ANN
R^2^	0.987	0.999	0.971	0.999	0.960	0.987
R^2^_Adj_	0.985	0.999	0.967	0.999	0.953	0.985
RMSE	0.040	0.004	1.72	0.127	2.35	1.31
MAE	0.030	0.003	1.42	0.096	1.83	1.11
STD	0.347	0.345	10.32	10.18	11.76	11.76
SEP	4.81	2.65	3.32	2.08	4.30	3.20

#### Neural interpretation diagram (NID)

**Fig. 5:**
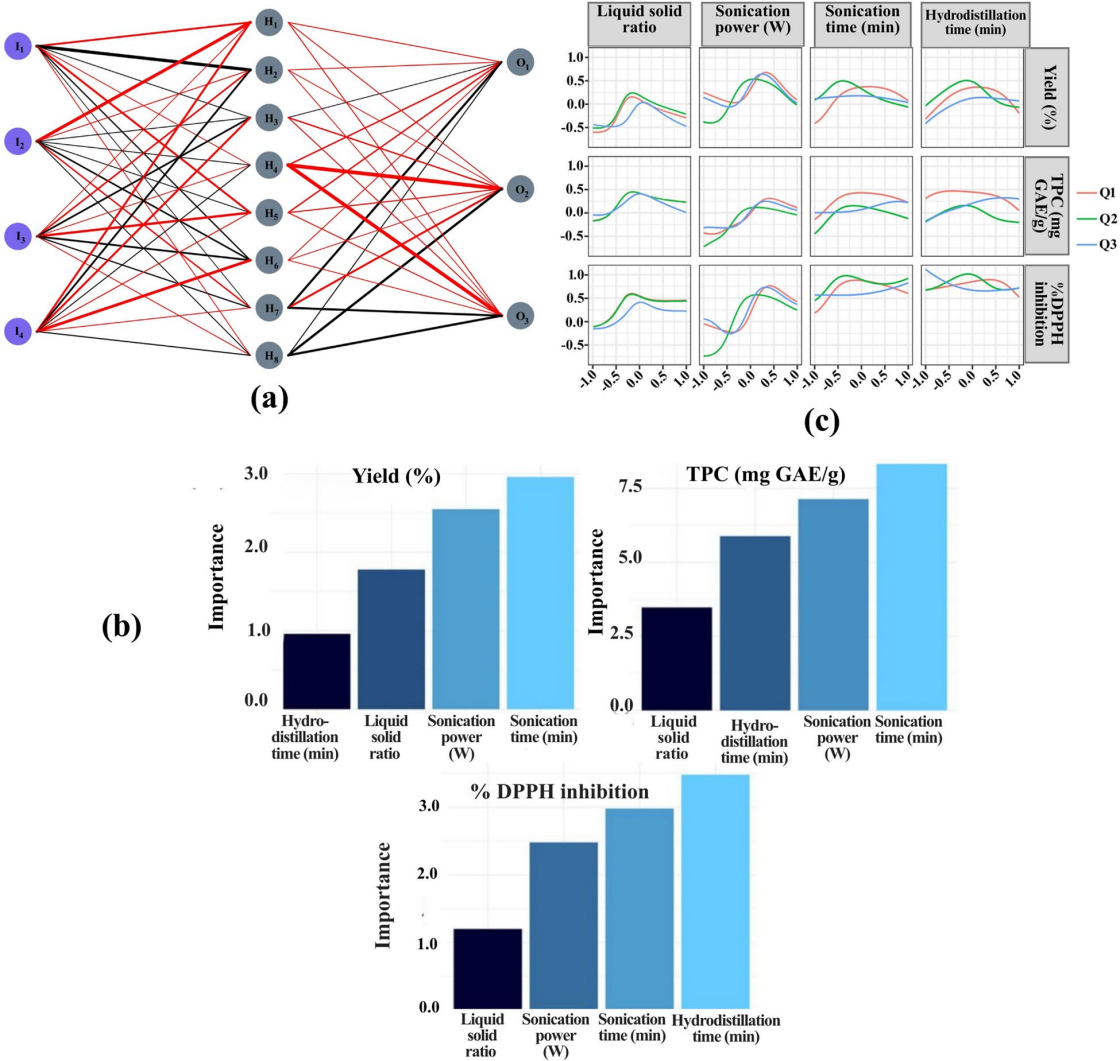
Artificial neural network modeling: **(a)** NID **(b)** Olden’s algorithm **(c)** Lek’s profile.

Figure 5a presents the NID which provides a qualitative visualization of how input variables influence the ANN model’s output, thereby enhancing the transparency of the decision-making process and helping to mitigate the model’s “black-box” nature^52^. NIDs have been widely applied in various research domains like extraction process optimization^32,45^, habitat suitability analysis^53^, parametric relationships in environmental modeling^27,90,91^, and others.

In this study, the NID is color-coded to indicate weight polarity (black for positive, red for negative), with line thickness representing weight magnitude. A positive effect is inferred when both input-hidden and hidden-output connections share the same color, either positive or negative^52^. For instance, inputs to H_1_ suggest a generally positive influence of all factors on responses, as indicated by the negative input-hidden and corresponding negative hidden-output weights. Conversely, inputs to H_2_ indicate that increasing LSR and HT while lowering SP and ST generally reduces responses, underscoring the critical role of sonication in the process. This aligns with our experimental data (Table 1), where run 25 (160 W, 40 min) achieved higher EO yield and DPPH activity than run 6 (80 W, 20 min), despite both having similar LSR (12:1) and HT (150 min). This is supported by previous studies on the synergistic effect of sonication and hydrodistillation in increasing extraction efficiency^21,24,25^. However, high yield was also achievable in lowered HT given that LSR and ST is increased (inputs to H_3_). Run 12, with a shorter HT of 90-min, yielded only 0.56% EO and showed lowered antioxidant capacity. However, increasing LSR and sonication effects helped compensate for the shorter HT, as seen in runs 19, 23, and 27, with significantly higher yields (0.86%, 0.69%, and 0.96%, respectively) despite similar HT durations. Increasing LSR may have enhanced solvent availability and mass transfer rates while reducing risk of local overheating leading to thermal degradation^92^. Meanwhile, raising SP and ST intensifies cell wall disruption and mass transfer through micro-mixing, enabling rapid release of EO components^66,93^.

Moreover, inputs to H_4_ suggest that reducing LSR and ST substantially reduces responses, reinforcing the pattern observed in inputs to H_3_. A similar trend was reported in the extraction of EO from bitter leaf^80^, further emphasizing the importance of these parameters in optimizing EO yield. Furthermore, inputs to H_6_ suggest that a substantial reduction in HT (−α) negatively affects responses, even when other parameters are at potentially optimal levels, as seen in Run 4. Similarly, inputs to H_8_ indicate that increasing SP and HT can improve responses despite lower ST, as observed in Run 5. This effect has also been reported in previous studies^93,94^. The presence of these conditional non-linear relationships indicates the limitations of traditional linear models like RSM, reinforcing the advantages of ANN in capturing such complex interactions^53^.

However, while NIDs are useful to provide valuable insights into the network’s weight distributions and interdependencies, they have certain limitations. Primarily, it captures only a static view of the trained model, omitting the dynamic weight adjustments that occur during the learning process. Additionally, because NID interpretations are largely qualitative, they benefit from the support of complementary quantitative methods such as variable importance estimation and sensitivity analysis. Integrating these techniques may enhance the reliability and interpretability of the model’s outcomes.

### Olden’s algorithm

Olden’s algorithm was employed to determine the relative importance of input variables in the ANN model by ranking their contributions to output predictions^54^. This analysis highlights the most influential factors governing the extraction process of BLEO, contributing to the model’s interpretability (Fig. 5b).

In Fig. 5b, all variables exhibited a generally positive effect on the responses, consistent with NID findings for inputs to H_1_. Moreover, sonication parameters had a significant influence in predicting yield, with ST having highest importance score, closely followed by SP. Interestingly, RSM model also predicted ST to have lower p value (< 0.0001) than SP (p value: 0.004), marking higher significance (Table 3). HT, while important, appeared to have the least influence on yield. The Olden plot for TPC also shows ST with the highest importance score while SP also significantly influences TPC, followed by HT and LSR. In contrast, the Olden plot for DPPH inhibition shows HT is the most important. ST and SP also contribute notably to DPPH radical scavenging activity, while the LSR has the least influence. These insights suggest that optimizing sonication parameters is crucial for maximizing EO yield^80^, whereas adjusting HT and LSR is key for enhancing phenolic content and antioxidant activity^68^.

### Sensitivity analysis

To further investigate the influence of SAHD process variables, Lek’s sensitivity analysis was performed on the ANN model (Fig. 5c). Unlike Olden’s method, which ranks variables based on their overall impact, Lek’s approach generates profile plots illustrating how predictions change across the range of a single input while holding others constant^55,95^. Specifically, when one variable is varied, all other input variables are held constant at predefined percentiles.

In Fig. 5c, Lek’s profile plots revealed a clear non-linear relationship between process variables and responses, where all three responses increased to a peak and then plateaued as the corresponding parameter increased, aligning with the findings from a previous study^96^. For BLEO yield, the plots suggested that slightly higher SP than the central point, combined with a shorter ST, may result in higher yield. The analysis also emphasized the stronger influence of LSR and HT on TPC compared to sonication parameters. HT and ST were identified as the most critical factors affecting DPPH radical scavenging activity, in agreement with Olden’s importance scores (Fig. 5b). The highest antioxidant activity was observed at moderate levels of HT and ST, while SP reached its optimum at slightly higher-than-moderate values^68^. Thus, Lek’s profile analysis helps define optimal parameter windows for efficient BLEO extraction.

### Comparison between RSM and ANN models

In our study on SAHD for BLEO extraction, we compared the performance of RSM and ANN models using statistical metrics such as R^2^, adjusted R^2^, MAE, RMSE, standard deviation (STD), and standard error of prediction (SEP). The results showed that ANN outperformed RSM across all evaluated metrics (Table 4), with R^2^ values approaching 1, indicating an almost perfect fit. In contrast, RSM showed slightly lower R^2^ values (0.987, 0.971, and 0.960 for yield, TPC, and DPPH inhibition, respectively), which still indicate good model performance.

**Fig. 6:**
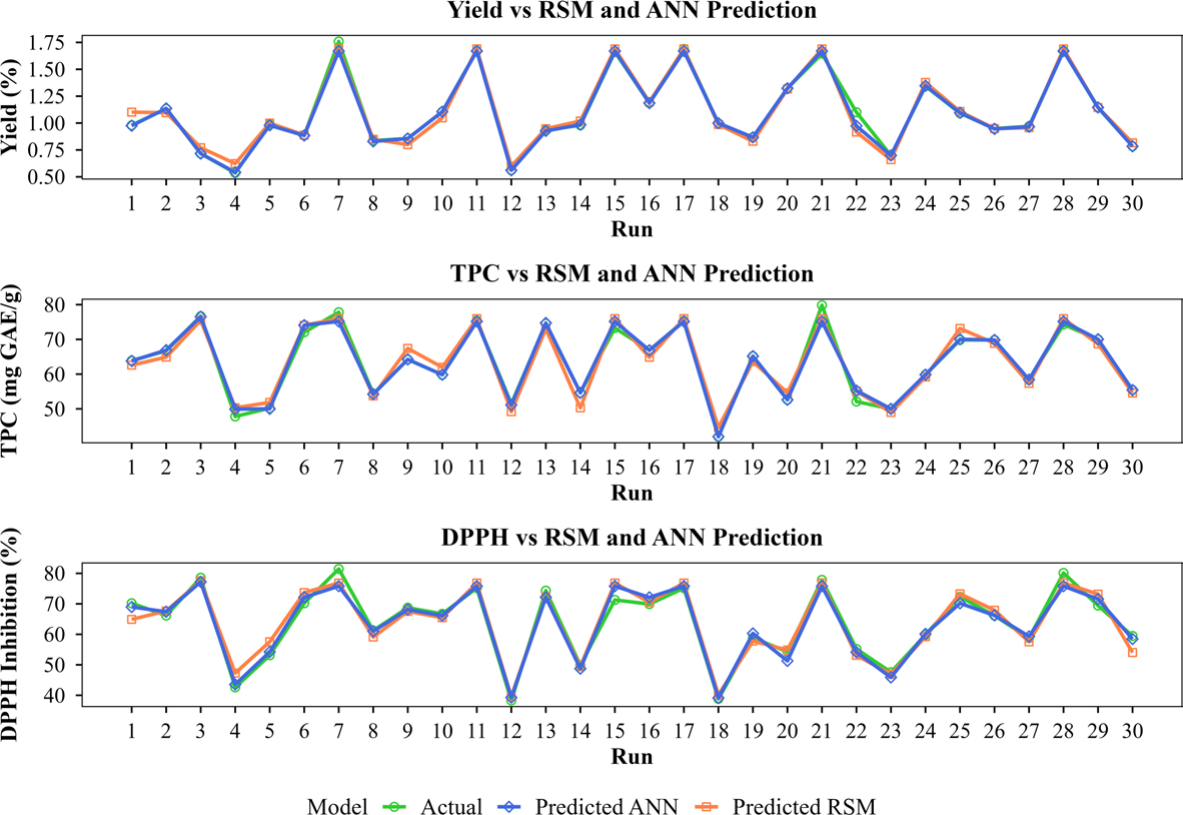
Experimental runs vs. RSM and ANN model predictions.

Figure 6 further supports these findings by illustrating the close alignment between experimental and predicted values. ANN provided a near-perfect fit for yield and more accurately captured trends in TPC. Both models performed comparably for DPPH inhibition, with only minor deviations.

While both models are effective, these results suggest that ANN demonstrated higher predictive accuracy with lower RMSE and MAE values, making it more reliable. This finding is consistent with previous studies that reporting that ANN outperforms RSM, particularly when modeling non-linear relationships^36,38,97,98^. The difference in performance stems from their underlying modeling approaches. RSM relies on predefined second-order polynomial equations, which may not fully capture complex non-linear interactions within dataset. Conversely, ANN models do not depend on predefined assumptions rather they learn intricate relationships through iterative training guided by data-driven weight adjustments and network architecture. This flexibility rendered ANN more effective in the present study, particularly for modeling the behavior of SAHD in relation to BLEO yield and antioxidant activity.

### Residual analysis

Recognizing that R^2^ and error metrics do not capture all aspects of model performance, we conducted a comprehensive residual analysis to assess model fit and identify potential biases. This approach provides a more granular view of model behavior, helping to detect underfitting or overfitting in specific data regions and ensuring that the underlying assumptions of regression models such as RSM and ANN are satisfied. In this study, residuals, defined as the differences between actual and predicted values, were analyzed for both RSM and ANN models (Supplementary Table 3) as well as for the ANN test data set (Supplementary Table 4).

In regression analysis, a key assumption is that the residuals are normally distributed^99^. In Supplementary Figs. 1 and 2, the residual plots revealed a random distribution of errors, suggesting a strong statistical relationship between the explanatory variables (LSR, SP, ST, HT) and the predicted responses. To further assess residual behavior, histograms and Q-Q plots were employed to evaluate normality (Supplementary Figs. 3–6). In all cases, residuals visually conformed to a normal distribution. To quantitatively assess this observation, Shapiro-Wilk and Kolmogorov-Smirnov tests were performed, yielding p-values greater than 0.05 for both the models and ANN test dataset (Table 5; Supplementary Table 5), thereby confirming that residuals were normally distributed.

**Table 5:** K-S test and Shapiro-Wilk test for normality of residuals.

	Kolmogorov-Smirnov^a^			Shapiro-Wilk		
	Statistics	df	significance	Statistics	df	significance
RSM-yield	0.123	30	0.200^*^	0.940	30	0.092
RSM-TPC	0.122	30	0.200^*^	0.968	30	0.494
RSM-DPPH	0.126	30	0.200^*^	0.956	30	0.241
ANN-yield	0.120	30	0.200^*^	0.957	30	0.256
ANN-TPC	0.152	30	0.075	0.952	30	0.193
ANN-DPPH	0.098	30	0.200^*^	0.979	30	0.805

* This is a lower bound of the true significance.

^a^ Lilliefors significance correction.

Another crucial assumption of regression analysis is the independence of residuals which refers to the condition where the error terms are not correlated with each other across observations^99^. To verify residual independence, Pearson and Spearman correlation coefficients were calculated between residuals and all dependent and independent variables (Supplementary Tables 6, 7). Almost all correlations were within the range of − 0.296 to 0.229 for residuals of both models, with p-values greater than 0.05, indicating weak, non-significant relationships. However, one exception was observed in the RSM model, where a moderate Spearman correlation (*r* = − 0.351) between TPC residuals and yield was detected. It was also statistically non-significant (*p* > 0.05) and therefore did not violate the assumption of residual independence. Furthermore, autocorrelation analysis, shown in Supplementary Fig. 7, revealed no visually significant serial correlation, this observation was further supported by the Durbin-Watson test, with coefficients close to 2 (Supplementary Table 8), supporting the assumption of independence.

Homoscedasticity assumes that residuals exhibit constant variance across all levels of the independent variables, a key requirement for ensuring valid inference in regression models^100^. Homoscedasticity was visually assessed through scale-location plots (Supplementary Fig. 8) and statistically evaluated using Levene’s test (Supplementary Table 9). No systematic trends were observed, supporting the assumption of homoscedasticity, with Levene’s test yielding p-values above 0.05. Additionally, the mean of the errors was close to zero (Supplementary Table 10), indicating unbiased predictions from both models.

Overall, rigorous residual analysis demonstrates that both RSM and ANN models provide reliable representations of the SAHD process, as they satisfy essential regression assumptions including normality, independence, homoscedasticity, and unbiased prediction behavior.

### Process optimization

In this study, two optimization approaches, RSM-DF and ANN-GA, were employed to optimize the SAHD extraction of BLEO (Table 6).

**Table 6:** Optimization results for RSM-DF and ANN-GA.

	I_1_	I_2_	I_3_	I_4_	Yield (%)		TPC (mg GAE/g)		DPPH inhibition (%)	
					Predicted	Actual	Predicted	Actual	Predicted	Actual
RSM-DF	10.90	127.59	31.24	129.92	1.66	1.64 ± 0.25	79.21	77.26 ± 0.96	80.82	78.22 ± 1.03
ANN-GA	9.89	141.73	26.83	123.86	1.68	1.67 ± 0.13	79.36	79.24 ± 0.82	82.16	81.54 ± 0.88

The RSM-DF method utilizes the desirability function for optimization, which combines the fitted model’s responses into a single overall desirability score, facilitating the identification of optimal conditions on the response surface. In contrast, genetic algorithms are population-based optimization techniques inspired by natural selection. Each candidate solution is represented as a chromosome, with characteristics encoded solution in genes. Through iterative processes such as selection, crossover, and mutation, the population evolves, progressively improving the overall fitness with each generation. As shown in Supplementary Fig. 10, starting from generation 100, the best, mean, and median fitness values stabilized, indicating successful convergence towards an optimal solution. The RSM-DF method identified optimal conditions as an LSR of 10.90:1, SP of 127.59 W, ST of 31.24 min, and HT of 129.92 min. Predicted values (1.66%, 79.21 mg GAE/g, 80.82%) from RSM-DF closely matched experimental outcomes for BLEO yield (1.64 ± 0.25%), TPC (77.26 ± 0.96 mg GAE/g), and DPPH radical scavenging activity (78.22 ± 1.03%). Similarly, the ANN-GA method optimized conditions to an LSR of 9.89:1, SP of 141.73 W, ST of 26.83 min, and HT of 123.86 min, yielding slightly higher accuracy in aligning predictions (1.68%, 79.36 mg GAE/g, 82.16%) with actual values (1.67 ± 0.13%, 79.24 ± 0.82 mg GAE/g, 81.54 ± 0.88%). Overall, the ANN-GA method outperformed RSM-DF, demonstrating closer agreement between predicted and experimental results across all measured parameters.

### GC–MS profiling

GC–MS analysis of the BLEO revealed a chemical profile dominated by linalool (47.37%), eugenol (18.34%), and cinnamaldehyde (16.45%), which together comprise approximately 82% of the total composition (Table 7). These compounds are recognized chemotypes in bay leaf and are primarily responsible for its characteristic aroma, bioactivity, and antioxidant potential. Terpenoids such as linalool, α-pinene, and β-caryophyllene contribute to the oil’s fragrance and therapeutic properties, while phenolic compounds like eugenol and methyleugenol enhance antioxidant activity. Minor constituents (1–10%) including methyleugenol and β-caryophyllene, and trace components (< 1%), such as β-pinene and cis-nerolidol, may contribute synergistically to the overall bioefficacy.

**Table 7:** GC/MS profiling of the extracted EO from bay leaf.

No.	Compound	Rel. Comp. HD (%)	^a^Retention index	^b^Retention index
1	α-Pinene	1.94	912	915
2	β-Pinene	0.064	944	945
3	α-Phellandrene	0.400	994	998
4	β- Phellandrene	0.190	1002	1002
5	**Linalool**	47.37	1081	1084
6	Cinnamaldehyde-linalool	0.030	1113	1115
7	**Cinnamaldehyde**	16.45	1168	1170
8	α-Terpineol	0.054	1198	1199
9	(z)-Cinnamaldehyde	trace	1221	1224
10	β-Terpineol acetate	trace	1344	1345
11	**Eugenol**	18.34	1377	1379
12	β-elemene	-	1385	1389
13	Methyleugenol	3.07	1394	1395
14	Beta-Caryophyllene	1.90	1420	1423
15	α-Humulene	0.840	1436	1439
16	Ethyl vanillin	trace	1466	1471
17	Trans-Cinnamyl acetate	2.19	1498	1501
18	Eugenolacetate	1.25	1527	1532
19	Germacrene	0.650	1533	1538
20	cis-Nerolidol	trace	1536	1539
21	Phenol	0.047	1549	1551
22	Spathulenol	0.987	1578	1579
23	Caryophyllene oxide	0.300	1583	1588
24	Ascabin	0.980	1846	1877
25	Hexadecanoic acid	0.490	1949	1961
	Total	97.54		

Trace: below 0.01%.

^a^ Retention index of each compound calculated in DB-1 column by retention time with that of n-alkanes (C8–C26).

^b^ Retention indices that refer to NIST Chemical Web Book.

Previous studies have reported considerable variation in the chemical composition of BLEO depending on geographic origin^101,102^. Samples from India and Nepal showed linalool as the major constituent, accounting for 50.40% and 32.18%, respectively, along with significant amounts of cinnamaldehyde and α-pinene^101^. Oil extracted from Munsyari, India, contained linalool (52.50%) and E-cinnamaldehyde (26.40%), with 1,8-cineole (4.20%) also present. In contrast, a sample from Hisar, New Delhi, reported cinnamaldehyde (44.89%) and trans-cinnamyl acetate (25.32%) as dominant, with linalool content as low as 0.44%^10^. Another study identified eugenol (45.28%), β-pinene (10.03%), and β-myrcene (9.73%) as key constituents^102^. These compositional differences highlight the influence of environmental conditions, genetic factors, and extraction methods on the phytochemical profile of BLEO. The chemical composition of BLEO in the current study reinforces its potential applications in therapeutic, flavoring, and preservative applications.

### Physico-chemical parameters

The physico-chemical characteristics of the extracted BLEO were analyzed to evaluate its quality and potential for industrial applications (Table 8). The oil exhibited a golden-yellow color and a characteristic pungent aroma, primarily attributed to its high eugenol content^103^. The specific gravity of BLEO at 25 °C was measured as 0.976 ± 0.014, which falls within the typical range for EOs. This value is comparable to that of lemongrass EO (0.98), while previous studies have reported lower specific gravity values for *Citrus limetta* peel oil (0.86) and *Citrus limon* peel oil (0.85)^104,105^.

**Table 8:** Physico-chemical properties of the extracted EO.

Parameters	Observations
Color	Yellow
Odor	Pungent
Specific gravity at 25 °C	0.976 ± 0.014
Acid value	2.02 ± 0.16 mg KOH/g
Iodine value	21.73 ± 1.25 g I_2_/100 g
Saponification value	141.39 ± 3.67 mg KOH/g
Ester value	29.82 ± 2.08 mg KOH/g

The acid value of the extracted EO was 2.02 ± 0.16 mg KOH/g, indicating low free fatty acid content and good oxidative stability^105,106^. This is comparable to *C. tamala* leaf EO, which has reported an acid value of 0.76 mg KOH/g, suggesting a long shelf life^88^. In contrast, higher acid values have been observed in other EOs, such as *Citrus macroptera* (4.17 mg KOH/g) and *Citrus sinensis* (3.71 mg KOH/g)^107,108^. The relatively lower acid value of BLEO thus reflects its enhanced stability under the applied extraction conditions.

In this study, the saponification value of BLEO was determined to be 141.39 ± 3.67 mg KOH/g. In contrast, a much higher saponification value of 300 mg KOH/g was reported for *C. tamala* EO from Nepal, likely due to regional and environmental differences influencing oil composition^88^. Interestingly, the saponification value of lemongrass EO (140.25 mg KOH/g) closely aligns with the present findings, suggesting similarities in fatty acid composition between BLEO and lemongrass oil^108,109^.

A relatively low iodine value of 21.73 ± 1.25 g I_2_/100 g was observed for BLEO, indicating a low degree of unsaturation and consequently a greater resistance to oxidative degradation^110^. In comparison, the iodine value of *C. tamala* EO has previously been reported as 36.20 g I_2_/100 g^88^. Significantly higher values have also been noted in other EOs, such as *Citrus sinensis* (82 g I_2_/100 g) and lemongrass (84.60 g I_2_/100 g)^105,107^. These comparisons highlight the superior oxidative stability of BLEO, attributable to its lower unsaturation level.

The ester value of BLEO was determined to be 29.82 ± 2.08 mg KOH/g. In comparison, a previous study on *Cinnamomum zeylanicum* oil reported a higher ester value of 46.00 mg KOH/g^111^. The relatively lower ester value of BLEO indicates enhanced storage stability and a longer shelf life, which may be attributed to the presence of short-chain fatty acids.

## Conclusion

This study successfully optimized the SAHD process for EO extraction from bay leaves using both RSM-DF and ANN-GA. The ANN-GA model demonstrated superior predictive accuracy and generalization capability. The optimal extraction conditions identified were a LSR of 9.89:1, SP of 141.73 watts, ST of 26.83 min, and HT of 123.86 min. Under these conditions, the BLEO yield reached 1.67 ± 0.13%, accompanied by high antioxidant activity, surpassing the performance of conventional hydrodistillation methods. Interpretability tools, including sensitivity analysis and Olden’s algorithm, provided valuable insights into the influence of individual process variables, enhancing transparency of the model. Model reliability was confirmed through residual diagnostics and experimental validation. GC–MS profiling and physico-chemical analysis verified the quality and industrial relevance of the optimized oil. However, the study was limited to laboratory-scale experiments and plant material from a single geographic location, which may affect the generalizability of the results. Future research should focus on scale-up validation, broader bioactivity screening, and comparative assessment with alternative green extraction technologies.

## Supplementary Information

Below is the link to the electronic supplementary material.

Supplementary Material 1

## Data Availability

Data will be made available on request from the corresponding author.
