# Supplementary material for: Optimizing sonication-assisted hydrodistillation of Cinnamomum tamala essential oil using response surface methodology and artificial neural network modeling
Source: Sci Rep. 2026 Mar 18;16:14107. doi: 10.1038/s41598-026-42869-2 (PMC13136388; doi:10.1038/s41598-026-42869-2)
Supplement: Supplementary file 1 — Supplementary Material 1 [file 41598_2026_42869_MOESM1_ESM.docx]

**Supplementary Table 1:** Test dataset for ANN modeling.

| **Run no.** | **Liquid-solute ratio (mL/g)** | **Sonication power (Watt)** | **Sonication time (min)** | **Hydro-distillation time (min)** | **Observed values** | | | **ANN predictions** | | |
| --- | --- | --- | --- | --- | --- | --- | --- | --- | --- | --- |
|  |  |  |  |  | **Yield (%)** | **TPC (mg GAE/g)** | **%DPPH inhibition** | **Yield (%)** | **TPC (mg GAE/g)** | **%DPPH inhibition** |
| **2** | 10 | 200 | 30 | 120 | 1.12 | 66.57 | 68.29 | 1.13 | 66.91 | 67.39 |
| **3** | 12 | 160 | 20 | 150 | 0.70 | 76.61 | 76.28 | 0.71 | 76.51 | 77.21 |
| **6** | 12 | 80 | 20 | 150 | 0.89 | 71.92 | 71.97 | 0.88 | 74.08 | 72.14 |
| **7** | 10 | 120 | 30 | 120 | 1.67 | 77.43 | 77.04 | 1.66 | 75.15 | 75.80 |
| **9** | 10 | 120 | 10 | 120 | 0.85 | 63.95 | 66.48 | 0.85 | 64.24 | 68.13 |
| **16** | 10 | 120 | 50 | 120 | 1.19 | 66.28 | 71.88 | 1.18 | 66.85 | 72.11 |
| **20** | 8 | 80 | 40 | 150 | 1.33 | 52.69 | 52.89 | 1.32 | 52.62 | 51.28 |
| **22** | 10 | 40 | 30 | 120 | 0.98 | 52.28 | 52.83 | 0.97 | 55.26 | 54.12 |
| **30** | 8 | 160 | 20 | 90 | 0.77 | 55.01 | 60.21 | 0.78 | 55.42 | 58.47 |

**Supplementary Table 2:** Evaluation of test dataset for ANN modeling.

|  | Yield | TPC | DPPH |
| --- | --- | --- | --- |
| R^2^ | 0.999 | 0.980 | 0.982 |
| RMSE | 0.008 | 1.47 | 1.22 |
| Adj R^2^ | 0.999 | 0.959 | 0.964 |

**Supplementary Table 3:** Residuals of RSM and ANN model predictions.

| Run | RSM | | | ANN | | |
| --- | --- | --- | --- | --- | --- | --- |
|  | **Yield** | **TPC** | **DPPH** | **Yield** | **TPC** | **DPPH** |
| 1 | −0.122 | 1.32 | 5.29 | 0.005 | 0.121 | 1.22 |
| 2 | 0.036 | 1.82 | −1.47 | −0.004 | −0.202 | −1.23 |
| 3 | −0.054 | 1.18 | 0.989 | −0.000 | 0.176 | 1.34 |
| 4 | −0.087 | −0.490 | −4.69 | −0.006 | −0.104 | −0.962 |
| 5 | −0.012 | −1.66 | −4.43 | 0.009 | 0.200 | −1.08 |
| 6 | −0.003 | 0.069 | −3.50 | 0.001 | 0.033 | −1.96 |
| 7 | −0.011 | −0.800 | −0.023 | 0.009 | 0.022 | 1.01 |
| 8 | −0.012 | 0.633 | 2.14 | 0.008 | 0.135 | 0.227 |
| 9 | 0.057 | −3.20 | 1.21 | 0.000 | −0.024 | 0.595 |
| 10 | 0.057 | −2.16 | 1.25 | 0.000 | −0.021 | 0.597 |
| 11 | −0.018 | −0.600 | −1.66 | 0.002 | 0.222 | −0.630 |
| 12 | −0.038 | 2.29 | −1.32 | 0.001 | 0.376 | −0.965 |
| 13 | −0.012 | 1.50 | 1.94 | 0.007 | −0.184 | 2.17 |
| 14 | −0.040 | 4.35 | 0.435 | −0.006 | 0.017 | 1.09 |
| 15 | −0.028 | −0.870 | −0.413 | −0.008 | −0.048 | 0.620 |
| 16 | −0.010 | 2.03 | −0.607 | 0.000 | 0.031 | −2.20 |
| 17 | −0.024 | −0.700 | −1.70 | −0.004 | 0.121 | −0.670 |
| 18 | 0.009 | −2.66 | −1.34 | −0.004 | −0.022 | −0.305 |
| 19 | 0.037 | 1.54 | 1.09 | 0.000 | −0.002 | −1.42 |
| 20 | 0.007 | −2.00 | −0.554 | −0.000 | −0.011 | 3.00 |
| 21 | −0.026 | −0.770 | −0.703 | −0.006 | 0.052 | 0.330 |
| 22 | 0.056 | 0.059 | 2.08 | −0.001 | −0.127 | 1.06 |
| 23 | 0.038 | 1.00 | 0.698 | −0.002 | −0.015 | 1.70 |
| 24 | −0.035 | 0.646 | 0.597 | −0.004 | 0.003 | −0.288 |
| 25 | −0.016 | −3.32 | −0.980 | −0.003 | −0.204 | 2.13 |
| 26 | 0.000 | 0.974 | −1.74 | 0.003 | 0.006 | −0.059 |
| 27 | 0.013 | 0.976 | 1.20 | 0.004 | −0.116 | −0.589 |
| 28 | −0.018 | −0.730 | −1.66 | 0.002 | 0.092 | −0.630 |
| 29 | −0.002 | 1.31 | −3.75 | −0.002 | −0.020 | −2.12 |
| 30 | −0.032 | 0.882 | 5.44 | 0.000 | 0.010 | 0.997 |

**Supplementary Table 4:** Residuals for ANN test dataset.

| **Run no.** | **Yield** | **TPC** | **DPPH** |
| --- | --- | --- | --- |
| **2** | 0.001 | 2.27 | 1.24 |
| **3** | −0.007 | 0.096 | −0.934 |
| **6** | 0.008 | −2.17 | −0.177 |
| **7** | 0.010 | 0.069 | 1.61 |
| **9** | −0.011 | −0.410 | 1.74 |
| **16** | 0.007 | −0.579 | −0.234 |
| **20** | 0.009 | −2.99 | −1.30 |
| **22** | −0.001 | −0.294 | −1.65 |
| **30** | −0.007 | −0.342 | 0.893 |





**Supplementary Fig. 1.** Residuals scatter plot of RSM and ANN model predictions.





**Supplementary Fig. 2.** Residuals scatter plot for ANN test set.





**Supplementary Fig. 3.** Residuals histogram of RSM and ANN model predictions.





**Supplementary Fig. 4.** Residual histogram for ANN test set.





**Supplementary Fig. 5.** Residuals Q-Q plot of RSM and ANN model predictions.





**Supplementary Fig. 6.** Residuals Q-Q plot for ANN test set.

**Supplementary Table 5:** K-S and Shapiro-Wilk test for ANN test set.

|  | **Kolmogorov-Smirnova** | | | **Shapiro-Wilk** | | |
| --- | --- | --- | --- | --- | --- | --- |
|  | **Statistic** | **df** | **Sig.** | **Statistic** | **df** | **Sig.** |
| **Res_test_yield** | 0.209 | 9 | 0.200* | 0.914 | 9 | 0.348 |
| **Res_test_TPC** | 0.252 | 9 | 0.105 | 0.905 | 9 | 0.283 |
| **Res_test_DPPH** | 0.168 | 9 | 0.200* | 0.918 | 9 | 0.379 |

**Supplementary Table 6:** Pearson correlation for RSM and ANN model predictions.

| **Measure** | **Variable** | **Liquid solid**  **ratio** | **Sonication**  **power** | **Sonication**  **time** | **Hydrodistillation**  **time** | **Yield** | **TPC** | **DPPH** |
| --- | --- | --- | --- | --- | --- | --- | --- | --- |
| **Res_RSM_yield** | Correlation | 0.059 | −0.100 | 0.056 | −0.274 | 0.023 | −0.106 | −0.058 |
|  | Significance | 0.758 | 0.599 | 0.768 | 0.143 | 0.903 | 0.577 | 0.762 |
| **Res_RSM_TPC** | Correlation | 0.045 | −0.177 | −0.045 | −0.042 | −0.296 | 0.001 | −0.135 |
|  | Significance | 0.813 | 0.350 | 0.814 | 0.826 | 0.112 | 0.997 | 0.478 |
| **Res_RSM_DPPH** | Correlation | 0 | 0 | 0 | 0 | −0.141 | −0.059 | 0.092 |
|  | Significance | 1 | 1 | 1 | 1 | 0.458 | 0.757 | 0.627 |
| **Res_ANN_yield** | Correlation | 0.229 | 0.024 | −0.055 | 0.011 | −0.095 | 0.055 | 0.193 |
|  | Significance | 0.223 | 0.900 | 0.774 | 0.954 | 0.616 | 0.774 | 0.306 |
| **Res_ANN_TPC** | Correlation | −0.202 | −0.180 | −0.282 | 0.080 | 0.018 | 0.008 | −0.046 |
|  | Significance | 0.284 | 0.340 | 0.130 | 0.675 | 0.927 | 0.965 | 0.810 |
| **Res_ANN_DPPH** | Correlation | −0.005 | −0.031 | 0.031 | 0.171 | 0.002 | −0.056 | 0.052 |
|  | Significance | 0.978 | 0.871 | 0.87 | 0.368 | 0.99 | 0.767 | 0.785 |

**Supplementary Table 7:** Spearman correlation for RSM and ANN model predictions.

| **Measure** | **Variable** | **Liquid solid ratio** | **Sonication**  **power** | **Sonication**  **time** | **Hydrodistillation**  **time** | **Yield** | **TPC** | **DPPH** |
| --- | --- | --- | --- | --- | --- | --- | --- | --- |
| **Res_RSM_yield** | Correlation | 0.108 | −0.105 | 0.188 | −0.251 | 0.029 | −0.187 | −0.198 |
|  | Significance | 0.571 | 0.581 | 0.320 | 0.181 | 0.880 | 0.322 | 0.294 |
| **Res_RSM_TPC** | Correlation | 0.056 | −0.184 | −0.095 | −0.085 | −0.351 | −0.030 | −0.178 |
|  | Significance | 0.769 | 0.331 | 0.617 | 0.656 | 0.058 | 0.875 | 0.348 |
| **Res_RSM_DPPH** | Correlation | 0.024 | −0.047 | 0.082 | −0.138 | −0.252 | −0.126 | −0.022 |
|  | Significance | 0.898 | 0.804 | 0.666 | 0.467 | 0.179 | 0.507 | 0.910 |
| **Res_ANN_yield** | Correlation | 0.204 | 0.065 | −0.085 | −0.073 | −0.129 | 0.071 | 0.167 |
|  | Significance | 0.280 | 0.735 | 0.655 | 0.700 | 0.498 | 0.711 | 0.377 |
| **Res_ANN_TPC** | Correlation | −0.097 | −0.136 | −0.284 | 0.141 | 0.049 | 0.185 | 0.197 |
|  | Significance | 0.610 | 0.475 | 0.128 | 0.457 | 0.798 | 0.328 | 0.296 |
| **Res_ANN_DPPH** | Correlation | −0.023 | 0.009 | 0.081 | 0.172 | −0.076 | −0.011 | 0.096 |
|  | Significance | 0.906 | 0.963 | 0.669 | 0.363 | 0.689 | 0.955 | 0.613 |





**Supplementary Fig. 7.** Residuals autocorrelation plot for RSM and ANN predictions.

**Supplementary Table 8:** Durbin Watson’s test for autocorrelation.

|  | **R** | **R^2^** | **Adjusted R^2^** | **Std. error** | **Durbin-Watson** |
| --- | --- | --- | --- | --- | --- |
| **Res_RSM_yield** | 0.479 | 0.229 | 0.069 | 0.038 | 1.56 |
| **Res_RSM_TPC** | 0.516 | 0.266 | 0.114 | 1.65 | 1.72 |
| **Res_RSM_DPPH** | 0.661 | 0.437 | 0.320 | 1.96 | 1.58 |
| **Res_ANN_yield** | 0.399 | 0.159 | −0.016 | 0.004 | 1.60 |
| **Res_ANN_TPC** | 0.534 | 0.285 | 0.136 | 0.119 | 2.42 |
| **Res_ANN_DPPH** | 0.672 | 0.452 | 0.338 | 1.08 | 1.91 |





**Supplementary Fig. 8.** Residuals scale location plot of RSM and ANN predictions.

**Supplementary Table 9:** Levine’s test for homoscedasticity of RSM and ANN models.

|  |  | Sum of squares | df | Mean square | F | Sig. |
| --- | --- | --- | --- | --- | --- | --- |
| Res_RSM_yield | Between groups | 0.001 | 2 | 0.001 | 0.304 | 0.740 |
|  | Within groups | 0.045 | 27 | 0.002 |  |  |
|  | Total | 0.046 | 29 |  |  |  |
| Res_RSM_TPC | Between groups | 5.95 | 2 | 2.97 | 0.970 | 0.392 |
|  | Within groups | 82.74 | 27 | 3.06 |  |  |
|  | Total | 88.69 | 29 |  |  |  |
| Res_RSM_DPPH | Between groups | 6.15 | 2 | 3.08 | 0.526 | 0.597 |
|  | Within groups | 157.89 | 27 | 5.85 |  |  |
|  | Total | 164.04 | 29 |  |  |  |
| Res_ANN_yield | Between groups | 0.000 | 2 | 0.000 | 0.220 | 0.804 |
|  | Within groups | 0.001 | 27 | 0.000 |  |  |
|  | Total | 0.001 | 29 |  |  |  |
| Res_ANN_TPC | Between groups | 0.079 | 2 | 0.040 | 2.70 | 0.085 |
|  | Within groups | 0.396 | 27 | 0.015 |  |  |
|  | Total | 0.476 | 29 |  |  |  |
| Res_ANN_DPPH | Between groups | 0.141 | 2 | 0.071 | 0.037 | 0.963 |
|  | Within groups | 51.04 | 27 | 1.89 |  |  |
|  | Total | 51.18 | 29 |  |  |  |

**Supplementary Table 10:** Mean of residual errors.

|  | RSM_yield | RSM_TPC | RSM_DPPH | ANN_yield | ANN_TPC | ANN_DPPH |
| --- | --- | --- | --- | --- | --- | --- |
| Mean error | −0.010 | 0.088 | −0.206 | 0.000 | 0.017 | 0.099 |


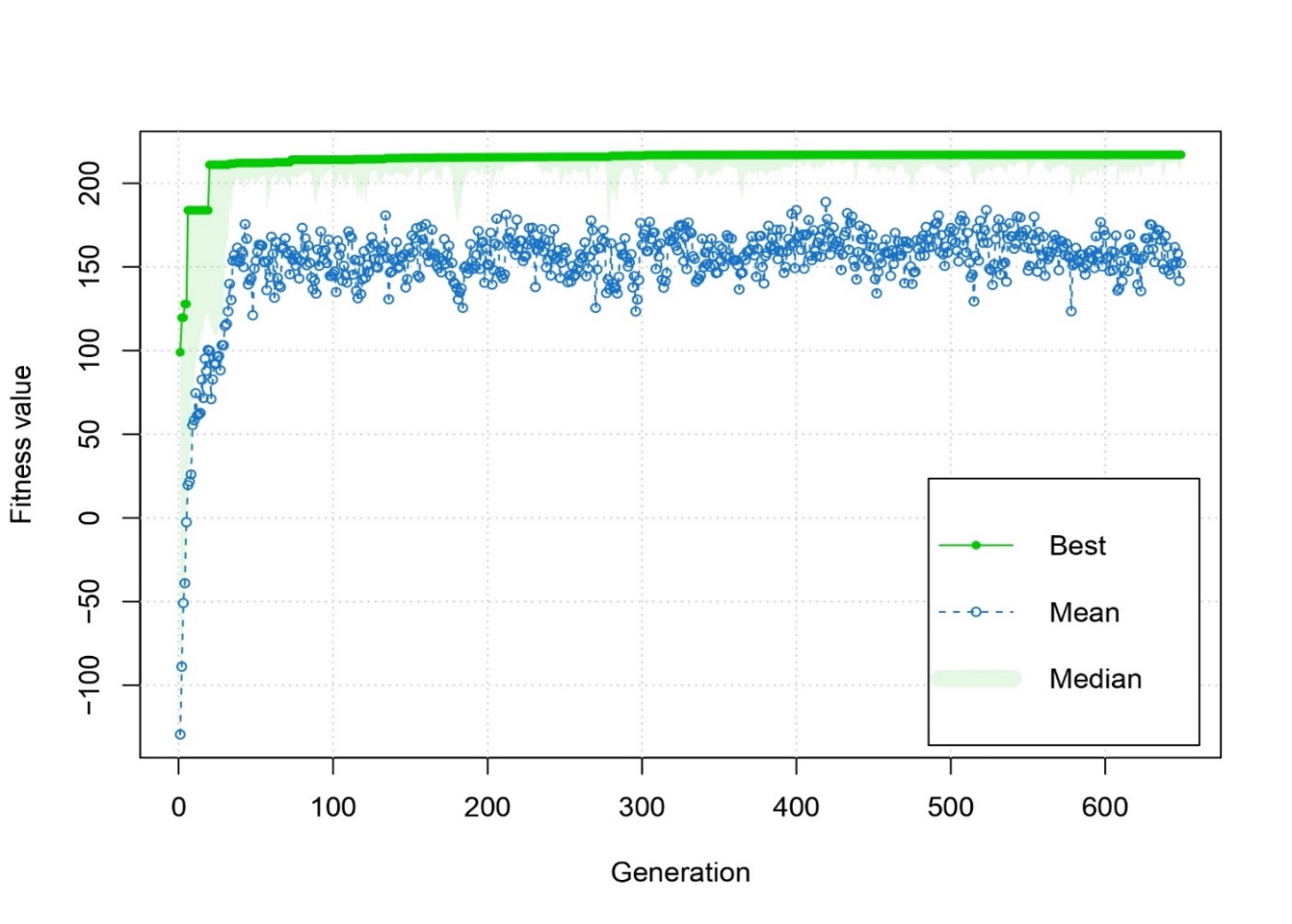


**Supplementary Fig. 9.** Genetic algorithm optimization of ANN model.
